# An Unlikely Trio: Profound Anemia, Elevated Lactate, and Riociguat

**DOI:** 10.7759/cureus.20997

**Published:** 2022-01-06

**Authors:** Mary Jo S Farmer, Harrison W Farber

**Affiliations:** 1 Medicine/Pulmonary and Critical Care Medicine, Baystate Medical Center/UMass Chan Medical School-Baystate, Springfield, USA; 2 Medicine/Pulmonary, Critical Care and Sleep Medicine, Tufts Medical Center/Tufts University School of Medicine, Boston, USA

**Keywords:** adempas, cteph, pah, riociguat, chronic thromboembolic pulmonary hypertension, pulmonary arterial hypertension, lactate, anemia, pulmonary hypertension

## Abstract

Riociguat (Adempas) is a stimulator of soluble guanylate cyclase approved to treat adult pulmonary arterial hypertension (PAH), World Health Organization (WHO) group 1 pulmonary hypertension (PH), and inoperable or persistent chronic thromboembolic pulmonary hypertension (CTEPH), WHO group 4 PH. Riociguat has been studied predominantly in WHO functional class II and III patients. In the pivotal Pulmonary Arterial Hypertension Soluble Guanylate Stimulator Trial 1 (PATENT-1) clinical trial, anemia was reported in 8% of patients; however, this anemia was typically very mild. We present a unique case of profound anemia and elevated lactate occurring in a patient taking riociguat for treatment of PAH.

## Introduction

Riociguat is the first-in-class oral drug that directly stimulates soluble guanylate cyclase, both independently of the endogenous vasodilator nitric oxide (NO) and in synergy with NO. Riociguat is approved to treat adult pulmonary arterial hypertension (PAH) (World Health Organization (WHO) group 1 pulmonary hypertension (PH)) to improve exercise capacity (placebo-adjusted mean increase in six-minute walk distance of 36 m within the riociguat group), improve functional class (21% in riociguat patients), and delay clinical worsening [1]. Riociguat is also indicated to treat adults with persistent/recurrent chronic thromboembolic pulmonary hypertension (CTEPH) (WHO group 4 PH) after surgical treatment or inoperable chronic thromboembolic pulmonary hypertension (CTEPH) to improve exercise capacity (placebo-adjusted mean increase in six-minute walk distance of 46 m within the riociguat group) and WHO functional class (33% in riociguat patients) [2]. Studies establishing effectiveness included the Pulmonary Arterial Hypertension Soluble Guanylate Stimulator Trial 1 (PATENT-1 trial) that predominantly included patients with WHO functional class II and III and etiologies of idiopathic or heritable PAH (61%) or PAH associated with connective tissue diseases (25%). In this trial, there was an 8% incidence of anemia in patients receiving riociguat. The mechanism of this anemia has not been determined, and the anemia was typically mild [3,4]. The World Health Organization classifies anemia by severity into mild (110 g/L to normal), moderate (80 g/L to 109 g/L), and severe anemia (less than 80 g/L) in adult males and adult nonpregnant females [5].

The most common causes of lactic acidosis include cardiogenic shock, severe heart failure, severe trauma, and sepsis, accounting for the vast majority of cases [6]. Causes of lactic acidosis have been divided into disorders with and without associated tissue hypoxia. Severe anemia, a less common cause of lactic acidosis, presumably results in elevated lactate due to decreased oxygen delivery to tissues and requires a hemoglobin level of less than 5 gm/dL [6].

Herein, we present an unusual case of profound anemia resulting in elevated lactate due to decreased oxygen delivery to tissues in a hemodynamically stable patient recently prescribed riociguat. This case was presented in part virtually at the American College of Chest Physicians Congress Italy on June 25, 2020.

## Case presentation

**Figure 1:**
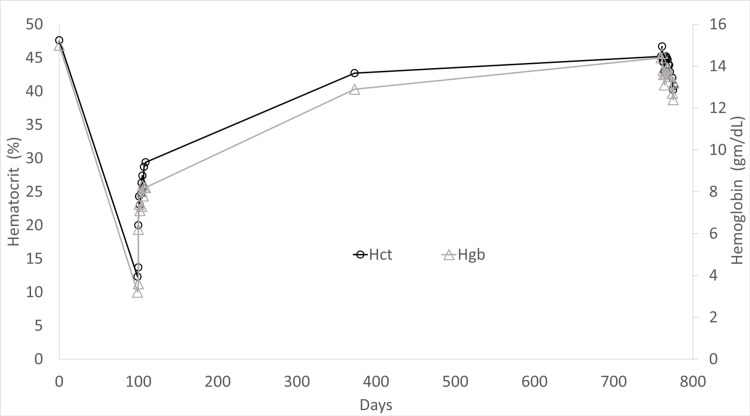
Hemoglobin and Hematocrit Hgb: hemoglobin; Hct: hematocrit. Day 0: baseline hemoglobin and hematocrit values.

A 56-year-old woman with lymphangioleiomyomatosis (LAM; cystic pattern), active smoking with 30 pack-year history, chronic hypoxia on 3 L/min supplemental oxygen, and pulmonary hypertension presented with worsening generalized weakness and inability to perform activities of daily living. Past medical history included T1cN1M0 estrogen-progesterone receptor-positive human epidermal growth factor receptor 2 negative infiltrating lobular carcinoma with ductal carcinoma in situ of left breast postmastectomy, axillary dissection and reconstruction (2002), and T3N0M0 estrogen-progesterone receptor-positive human epidermal growth factor receptor 2 negative infiltrating ductal carcinoma of right breast postmultiple reconstructions (2015) on anastrozole. Three months prior to presentation, echocardiogram estimated pulmonary artery systolic pressure at 90 mmHg and demonstrated severe right ventricular dilation with severe tricuspid regurgitation. Ventilation/perfusion scan demonstrated multiple mismatched perfusion defects in the right midlung and lung apices with high probability for pulmonary embolus (PE) and/or chronic thromboembolic pulmonary hypertension (CTEPH). Right heart catheterization demonstrated precapillary PH (Table 1). Pulmonary angiogram demonstrated extensive chronic thrombotic disease. The patient refused surgical evaluation and was treated as a combination of group 1 PH (prior daily cocaine use), group 3 PH (hypoxia), group 4 PH (CTEPH), and possible group 5 PH (LAM) with riociguat and apixaban. Hemoglobin (Hgb) was 15 g/dL and hematocrit (Hct) 47.6% (Figure 1) (day 0 baseline Hgb and Hct) before beginning treatment, not suggestive of anemia.

**Table 1:** Right Heart Catheterization Data

Parameter	Patient value	Normal value
Right atrial pressure	11/9 (7) mmHg	2-6 mmHg
Pulmonary artery pressure	60/12 (39) mmHg	9-18 mmHg mean
Pulmonary capillary wedge pressure	18/14 (12) mmHg	6-12 mmHg
Pulmonary vascular resistance (Fick)	412 dynes-sec/cm^5^	< 250 dynes-sec/cm^5^
Pulmonary vascular resistance (Thermodilution)	284 dynes-sec/cm^5^	
Cardiac output (Fick)	3.74 L/min-m^2^	4.0-8.0 L/min

On the day of presentation, the patient denied worsening shortness of breath, chest pain, abdominal pain, hematochezia, melena, bright red blood per rectum, or consistent nonsteroidal anti-inflammatory drug (NSAID) use. Vital signs were temperature 99.7 degrees F, pulse 93 beats per minute, respiratory rate 20 breaths/minute, blood pressure 116/71, and oxygen saturation 98% on 3-6 L/min supplemental oxygen. Physical examination revealed fine bilateral crackles on lung auscultation, a loud split S2 heart sound, and patient alert and oriented to person, place, and time with cranial nerves II-XII intact, motor strength proximal right upper extremity 5/5, distal right upper extremity 5/5, proximal left upper extremity 4/5, distal left upper extremity 4/5, right lower extremity 5/5, left lower extremity 5/5, sensation in right upper extremity, left upper extremity, right lower extremity, left lower extremity, facial, and trunk all normal to touch, normal bilateral finger to nose coordination, normal speech, and Glasgow coma scale 15. Laboratory evaluation was notable for severe anemia with hemoglobin 3.2 g/dL (female: 11.7-16 g/dL) and hematocrit 12.3% (female: 36%-48%) (Figure 1) (day 99 from baseline). Lactate was elevated at 12.5 mmol/L (0.93-1.65 mmol/L) with bicarbonate 17 mmol/L (22-29 mmol/L), anion gap 27 (4-17), and venous pH 7.29. Iron studies were suggestive of iron deficiency anemia with total iron-binding capacity 544 µg/dL (262-474 µg/dL), ferritin 30 ng/mL (females: 20-200 ng/mL), iron level 35 µg/dL (females: 26-170 µg/dL), and iron saturation 6% (10%-45%). Peripheral blood smear showed markedly microcytic and hypochromic red blood cells (RBCs). Lactate dehydrogenase was elevated at 607 units/L (140-333 IU/L) with mild indirect bilirubinemia 1.9 mg/dL (0.2-0.8 mg/dL), normal haptoglobin, and negative Coombs test. CT of the abdomen pelvis did not demonstrate a retroperitoneal bleed. Of note, colonoscopy eight years prior revealed external hemorrhoids and a tubular adenoma that was removed. Patient did not have repeat colonoscopy at five years as advised. Heme occult stool was positive, thought to be related to known hemorrhoids and intermittent although not active NSAID use when discussed with Hematology and Gastroenterology consultants.

During the current admission, the patient received four units of packed RBCs. Posttransfusion hemoglobin increased appropriately to 8.3 g/dL and hematocrit 27.4% (Figure 1) (day 105 from baseline). Lactate decreased to 1.7 mmol/L. Repeat hemoglobin and hematocrit were stable four days posttransfusion. MRI of the brain was reviewed with Neurology consultant and demonstrated a moderate sized area of restricted diffusion involving the right parietal lobe including the right parietal operculum and the right posterior insula cortex suggesting an acute infarct of the right middle cerebral artery territory, consistent with left upper extremity weakness. Apixaban was discontinued for two weeks to avoid hemorrhagic conversion per Neurology consultant. Hematology consultant recommended discontinuation of riociguat due to severe anemia attributed in part to iron deficiency with otherwise unidentified mechanism potentially related to riociguat. Since hemoglobin stabilized after discontinuation of riociguat and following blood transfusion, Gastroenterology consultant arranged for nonemergent outpatient colonoscopy. Unfortunately, the patient canceled the procedure due to anxiety and refused to leave her home during the COVID-19 pandemic. Repeat echocardiogram demonstrated a severely dilated right ventricle, reduced right ventricular systolic function, and estimated pulmonary artery systolic pressure of 67 mmHg. The patient was transitioned to sildenafil to treat pulmonary hypertension during hospitalization, and apixaban was restarted prior to discharge. Hemoglobin remained stable for approximately nine months after discontinuation of riociguat (hemoglobin, 12.9 g/dL and hematocrit, 42.7%) (Figure 1) (day 373 from baseline).

Posthospital discharge, the patient had several telemedicine appointments during the COVID-19 pandemic. She denied worsening shortness of breath, changes in bowel habits, or blood in the stool. Sildenafil was continued. During a hospital admission for volume overload 22 months after discontinuation of riociguat, hemoglobin and hematocrit were 13.5 g/dL and 43.9%, respectively (Figure 1) (day 771 from baseline). 

## Discussion

The gradual decline in hemoglobin to a severe level occurring during the three-month period of riociguat administration recorded in this case is to our knowledge a rare occurrence. Hemoglobin subsequently remained normal following blood transfusion and discontinuation of riociguat in this patient. The actual mechanism by which riociguat causes anemia which is usually mild remains to be identified, although expert opinion has proposed this anemia could be due to vasodilator effects which could result in bleeding from unidentified arteriovenous malformations or telangiectasias.

Elevated lactate associated with hemodynamic stability and normal vital signs supports a gradual decline in hemoglobin with steady compensation rather than an acute phenomenon. Given that hemoglobin is an important component of oxygen delivery, this phenomenon can be explained by understanding the critical concepts of oxygen delivery (DO_2_), oxygen consumption (VO_2_), and oxygen extraction ratio (O_2_ER) [7].

Oxygen extraction ratio (O_2_ER) is the ratio of oxygen consumption (VO_2_) to oxygen delivery (DO_2_) and normally ranges between 22% and 30%. Oxygen delivery (DO_2_) is the total amount of oxygen delivered to the tissues per minute, irrespective of the distribution of blood flow, normally approximately 1 L/min. DO_2_ is determined by arterial oxygen content (CaO_2_), of which hemoglobin is an important component, and cardiac output (CO). Oxygen consumption (VO_2_) is the total amount of oxygen removed from the blood due to tissue oxidative metabolism per minute, normally approximately 250 mL/min [8].

**Figure 2:**
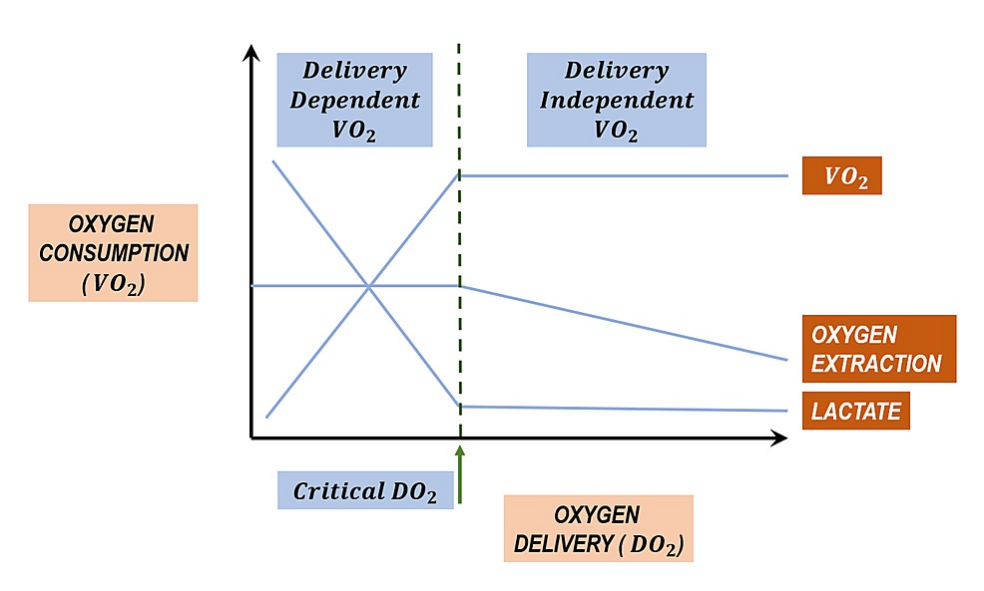
Relationship Between Oxygen Delivery (DO2) and Oxygen Consumption (VO2)

DO_2_ is more than adequate to meet VO_2_ and maintain aerobic metabolism under resting conditions with normal distribution of cardiac output. Initially, as VO_2_ increases with increasing metabolic demands or DO_2_ diminishes, O_2_ER rises to maintain aerobic metabolism and consumption remains independent of delivery. The constant relationship between VO_2_ and DO_2_ maintains optimum function. The “critical level” at which this relationship is lost leads to tissue dysoxia and a subsequent increase in lactate production (Figure 2) [8]. While normal O_2_ER is approximately 25%, critical DO_2_ (cDO_2_) is approximately 70%. Each tissue/organ has its own cDO_2_, for example, cardiac O_2_ER ≥ 60%, hepatic O_2_ER = 45%-55%, and renal O_2_ER ≤ 15%. The higher the O_2_ER for a given tissue, the greater the dependence on DO_2_ [7,9]. O_2_ER increases as the body tries to compensate by increasing cardiac output when hemoglobin decreases. Particular to this case, hemoglobin decreased to such a low level meeting the criteria for severe anemia resulting in decreased oxygen delivery to tissues. This mechanism explains the elevated lactate in the setting of stable hemodynamics recorded in this patient and also explains the decline in lactate as hemoglobin was corrected with RBC transfusion and oxygen delivery improved.

## Conclusions

In the pivotal clinical trial PATENT-1 for PAH, riociguat, a stimulator of soluble guanylate cyclase, was associated with an 8% incidence of anemia. In this case, the severely reduced hemoglobin and elevated lactate were associated with administration of riociguat, since no other entity was determined and there was long-term improvement and stabilization after its discontinuation. The exact mechanism of the profound anemia recorded in this case and the mild anemia typically associated with riociguat remain to be identified by further research. As such, we suggest periodic monitoring of hemoglobin and hematocrit in patients treated with riociguat. Also, in this patient, the physiology of critical oxygen delivery and uptake including oxygen extraction ratio in the setting of profound anemia supports the phenomenon of lactate production without evidence of hemodynamic decompensation.
